# Oxidative Stress and Gut Microbiome in Inflammatory Skin Diseases

**DOI:** 10.3389/fcell.2022.849985

**Published:** 2022-03-07

**Authors:** Qingrong Ni, Ping Zhang, Qiang Li, Zheyi Han

**Affiliations:** ^1^ Department of Dermatology, Air Force Medical Center, Fourth Military Medical University, Beijing, China; ^2^ Department of Gastroenterology, Air Force Medical Center, Fourth Military Medical University, Beijing, China

**Keywords:** inflammatory skin diseases, oxidative stress, gut microbiome, gut-skin axis, anti-oxidant therapies

## Abstract

Oxidative stress plays a dominant role in inflammatory skin diseases. Emerging evidence has shown that the close interaction occurred between oxidative stress and the gut microbiome. Overall, in this review, we have summarized the impact of oxidative stress and gut microbiome during the progression and treatment for inflammatory skin diseases, the interactions between gut dysbiosis and redox imbalance, and discussed the potential possible role of oxidative stress in the gut-skin axis. In addition, we have also elucidated the promising gut microbiome/redox-targeted therapeutic strategies for inflammatory skin diseases.

## Introduction

Oxidative stress acts as the essential regulator in the biological components, which has been noted that it could mediate the pathological progression of inflammatory skin diseases. Several studies showed that endogenous and exogenous modulatory factors such as amounts of biochemical components (oxygen, nitrogen, and sulfur) might result in oxidative stress reactions. Additionally, these active substances are called reactive species, mainly including reactive oxygen species (ROS), reactive nitrogen species (RNS), and reactive sulfur species (RSS), which have made stimulating effects on the progress of cell metabolism (Bourgonje et al., 2020). In detail, reactive species participate in a variety of oxidative signaling pathways, such as MAPK/AP-1, NF-κB, JAK-STAT, Nrf-2, PI3K/AKT, and TLR-mediated signaling, modifying cytochrome thiols, and then regulate the function of enzymes, transcription factors, and other proteins (Sies and Jones, 2020; Forman and Zhang, 2021). ROS and RNS can be produced in the skin during the procedure of respiration exposed to pollutants, toxins, or ultraviolet irradiation (D'Autréaux and Toledano, 2007; Willems et al., 2015; Zhang et al., 2016). ^1^O_2_ is the first ROS produced by the skin, rapidly metabolized to produce superoxide radicals (O_2_⋅^−^), hydrogen peroxide (H_2_O_2_), and ⋅OH. ⋅NO is one of the RNS produced by NO synthase (NOS) in UVB-irradiated or inflamed skin (Deliconstantinos et al., 1996). RSS has consisted of hydrogen sulfide (H_2_S), persulfide (RS_n_SH, n ≥ 1), polysulfide (H_2_S_n_, n ≥ 2), sulfur dioxide (SO_2_), carbon disulfide *et al.* Endogenous H_2_S is produced by l-cysteine catalyzed by cystathionine-*γ*-lyase (CSE) and cystathionine-*β*-synthase (CBS), while produced by *β*-mercaptopyruvate pyruvic acid in the presence of 3-mercaptopyruvate sulfotransferase (3-MST) in mitochondria(Kutz et al., 2015; Meng et al., 2018). At present, RSS is considered to participate in various pathological and physiological processes of skin diseases(Kutz et al., 2015; Goren et al., 2019; Yang et al., 2019; Xu et al., 2021; Zhou et al., 2022). Meanwhile, there have been cells have a variety of antioxidant mechanisms in cells to combat oxidative stress. Long-term antioxidant responses, which contain activation of pro-survival gene expression programs (e.g., Nrf2) and DNA damage repair (e.g., ATM and p53) or cell death correlated program (e.g., NF-κB and p53), are the crucial part of cell defense against stress (Martindale and Holbrook, 2002; Ray et al., 2012). The short-term antioxidant responses help to alleviate acute cell damage caused by oxidative stress and stabilize cell redox potential. These responses are activated by antioxidant cell systems that contain enzymes (e.g., superoxide dismutase, catalase, and glutathione-peroxidase) and non-enzymatic antioxidants such as vitamins and reduced glutathione (GSH) (Finkel and Holbrook, 2000; Chen et al., 2012; Kuehne et al., 2015).

The gut microbiome is presented in the gastrointestinal tract, including bacteria, viruses, archaea, fungi, and genetic material (Lederberg, 2001). Microbiome and related small molecule metabolites play essential roles in host immune maturation and dynamic balance (Brühl and Küstner, 1972; Dodd et al., 2017). The abnormal gut microbiome changes are closely related to the occurrence and development of inflammatory diseases such as inflammatory bowel disease (Cohen et al., 2019). Recent studies have shown that the gut microbiome, through their production of metabolites, could induce local and systemic inflammation, regulate the function of extra-intestinal organs such as the brain (Morais et al., 2021), joint (Zaiss et al., 2021), liver (Tranah et al., 2021) and skin *et al.*(Ni et al., 2020) by modulating the production and catabolism of metabolites, and participate in the occurrence and progression of diseases. Respectively, the concepts of “gut-brain axis,” “gut-joint axis,” “gut-liver axis,” and “gut-skin axis” were put forward. Individually, several studies have made further investigation and indicated that the indispensable interactions of oxidative stress and gut microbiome suggested that the cross-talk between redox imbalance and gut dysbiosis may display critical effects on the gut-skin axis.

In this review, the dramatic role of oxidative stress and gut microbiome in the progression of inflammatory skin diseases will be summarized. Moreover, the close association between oxidative stress and gut microbes will be delivered. All in all, the significance of its relationship and promising therapeutic strategies for inflammatory skin diseases by targeting gut microbiome/redox will also be elucidated.

## Oxidative Stress and Inflammatory Skin Diseases

### Role of Reactive Species in Skin

It is well known that several kinds of oxidative stress-induced diseases and abnormal biological components could be modulated by reactive species-associated inflammation and cell death (Sies and Jones, 2020). Meanwhile, the reactive species in the skin are in an unstable state to make it too ‘active.’ Among different subtypes of ROS, O_2_⋅^−^ is synthesized by NADPH oxidase (NOX) and Xanthine oxidase (XO). Increased NADPH oxidase activity of neutrophils and macrophages subsequently produce large amounts of O_2_⋅^−^, which may contribute to the cytotoxic reactions during inflammatory skin diseases (Ryu et al., 2019). Moreover, amounts of O_2_⋅^−^ mediated by NOX could participate in modulating differentiation and proliferation of epidermal keratinocyte and fibroblasts (Martínez-Navarro et al., 2020). The XO could also be found in epidermal keratinocytes and endothelial cells. ⋅NO is synthesized by NOS existing in keratinocytes, fibroblasts, and endothelial cells (Abbas et al., 2016). Active sulfur compounds (RSS) have more robust antioxidant activity than mercaptan. Three H_2_S synthases are identified in the skin, including CSE, CBS, and 3-MST. It has been reported that CSE and 3-MST are highly expressed in the human microvasculature (Kutz et al., 2015). CSE is also expressed in hair follicle keratinocytes and basal keratinocytes of the neo-epidermis at the wounded area. Furthermore, CSE-derived H_2_S can enhance the expression of early keratinocyte differentiation markers cytokeratin 10 (CK10) and involucrin (IVN) in cultured human keratinocytes (Goren et al., 2019). Yang et al. found that H_2_S attenuates NETosis and primes diabetic wounds to heal through blockage of ROS-mediated MAPK ERK1/2 and p38 activation (Yang et al., 2019). Zhou et al. found that YB-1 (a DNA-binding protein) promotes splicing of pri-miR-192 to mediate the proangiogenic effects of H_2_S(Zhou et al., 2022). These studies suggested the potential therapeutic effects of RSS on oxidative stress. Different reactive species can interact with each other. O_2_⋅^−^ forms ONOO- in the presence of a large amount of ⋅NO, and then break down into ⋅NO_2_ and ⋅OH. ⋅NO, ONOO-and ⋅NO_2_ nitrify the tyrosine residues of proteins to form nitro-tyrosine, which inhibit phosphorylation signaling (Bourgonje et al., 2020).

### Role of Oxidative Stress in Inflammatory Skin Diseases–Reactive Species as Mediators

Oxidative stress plays a vital role in the pathophysiological process of inflammatory skin diseases. Redox balance disorders induced by sustained exposure to reactive species accelerate the severe inflammation and cell death in the skin. The role of oxidative stress in several inflammatory skin diseases will be well discussed.

### Atopic Dermatitis

Overproduction of ROS plays a crucial role in the pathogenesis of atopic dermatitis (AD). A high level of ROS was observed in skin biopsies from AD patients (Sapuntsova et al., 2011). Meanwhile, up-regulated oxidative stress biomarkers, including NO, malondialdehyde (MDA), and 8-hydroxydeoxyguanosine (8-OHdG), have also been found in serum and urine of AD patients (Tsuboi et al., 1998; Omata et al., 2001; Tsukahara et al., 2003). A recent study from 56 children diagnosed with atopic dermatitis showed significantly higher lipid hydroperoxide and myeloperoxidase levels. In comparison, a lower serum level of the total antioxidant potential value indicates AD might be associated with increased oxidative stress reactions and decreased antioxidant defense (Simonetti et al., 2021). An observational study recruiting 31 children with AD demonstrated that the mean and total sulfhydryl concentrations were lower than those in the control group (*p* = 0.012; 0.047), while the average concentration of disulfide, SS/SH, SS/total SH, SH/total SH were significantly higher than those of healthy infants (*p* < 0.05), which suggested that the higher antioxidant defense level of AD infants (Karacan et al., 2020). The environment is a known source of oxidative stress. Many kinds of pollutants in the air may cause oxidative stress and lead to skin barrier dysfunction, inflammation, and immune disorders. Environmental pollutants, such as cigarette smoke bind to aryl hydrocarbon receptor (AhR) and induce the production of ROS and inflammatory factors. Previous studies have shown increased levels of 8-OHdG, a DNA oxidation marker, in AD children exposed to short-term ultrafine particles (Song et al., 2013). *Staphylococcus aureus* is the dermal colonization bacterium from AD patients. Notably, bacterial-related enzymes can combine AhR and produce ROS (Furue, 2020). Furthermore, evidence showed that oxidative stress might induce the poor progression and development of AD. One clue is that oxidative stress-related signal pathways such as the NF-κb pathway can induce the production of many inflammatory cytokines (such as IL-6, IL-8, IL-9, and IL-33) (Yao et al., 2011) and aggravate skin inflammation. The other clue is that current animal studies have proved that the oxidation-reduction imbalance in AD skin enhanced Th2 polarization, worsening the disease (Ahn, 2014).

### Psoriasis

Up-regulated ROS were found in the keratinocyte, fibroblasts, and neutrophils of the skin in psoriasis patients (Khmaladze et al., 2014). It has been reported that NOX4, which is expressed in dermal fibroblasts, is essential for keratinocytes proliferation (Barygina et al., 2019b). Moreover, ROS was involved in the neutrophil chemotaxis (Hoffmann et al., 2018; Aksoy and Kirmit, 2020). Vegfors et al. reported that ROS could induce the expression of psoriasis-associated angiogenic factors, vascular endothelial growth factor (VEGF), heparin-binding epidermal growth factor-like growth factor, matrix metalloproteinase one, and thrombospondin 1 (Vegfors et al., 2016). ROS-mediated oxidative stress activates various redox signaling pathways in psoriasis, including NF-κB and MAPK (Zhou et al., 2009), thus activating Th1 and Th17 secreting cytokines, finally causing the proliferation of keratinocytes. Barygina et al. also found that low dose cytokines can reduce oxidative stress in primary lesional fibroblasts of psoriasis (Barygina et al., 2016). Moreover, anti-TNF-α is therapy probably associated with normalization of NADPH oxidase activity in psoriasis patients (Barygina et al., 2013). Furthermore, Sirtuin 1 (Sirt 1) can protect against oxidative stress-induced apoptosis in fibroblasts via modulation of MAPK signaling in psoriasis (Becatti et al., 2018). As a complex disease with inflammation and oxidative stress, oxidative stress in psoriasis may have complex cross-talk mechanisms with metabolism and cytokines. Glycolytic reprogramming is triggered in macrophages and dendritic cells (DCs) following acute activation of pathogen-associated molecular patterns (especially Toll-like receptor), and then results in altered mitochondrial function, increased reactive oxygen species (ROS) production, and elevated secretion of proinflammatory cytokines (Tannahill et al., 2013; Lampropoulou et al., 2016). Mogilenko et al. found that high-fatty acids enhance Toll-like receptor-mediated innate activation by inhibiting hexokinase, thus impairing glycolysis reprogramming, leading to mitochondrial adaptation disorder, and increasing the production of mitochondrial reactive oxygen species (mtROS) (Mogilenko et al., 2019). The result accentuated the unfolded protein response (UPR) and induced the production of IL-23. Mizuguchi *et al.* also showed that mtROS-dependent IL-1β is involved in exacerbating psoriatic inflammation(Mizuguchi et al., 2021).

ROS also participates in lipid peroxidation in psoriasis. Unsaturated fat oxidation induced by ROS causes the production of MDA (Pietrzak et al., 2010). Some studies have found elevated MDA and NO in serum, reduced SOD (superoxide dismutase, a group of enzymes that catalyze the dismutation of O_2_⋅^−^ to O_2_ and H_2_O_2_), and total antioxidant capacity (TAC) in serum of psoriasis patients. Furthermore, Sorokin *et al.* found that assaying oxidation-modification of lipids revealed a significant association with oxidized LDL and oxHDL in psoriasis patients (Sorokin et al., 2018). ROS-mediated lipid peroxidation in the skin of psoriasis induced oxidized LDL (ox-LDL) (Honda and Kabashima, 2019) and caused the activation of phospholipase A2, exacerbating local inflammation. Meanwhile, lipid peroxidation also activated cGMP, leading to excessive epidermal proliferation in psoriasis (Aksoy and Kirmit, 2020). A recent study showed that ROS is involved in cell death in psoriasis. The excessive activation of PARP1 was responded to ROS-induced DNA damage and resulted in parthanatos cell death (Martínez-Morcillo et al., 2021).

Meanwhile, evidence suggested that RNS plays an essential role in the pathogenesis of psoriasis. Researches confirmed that CD11c (+) cells (a type of DCs), which are the major cell types in the skin lesions of psoriasis, are the sites for the expression of inducible NOS (Lowes et al., 2005). Moreover, it has been known that the locus containing the inducible nitric oxide synthase (NOS2) gene is associated with psoriasis susceptibility (Lowes et al., 2005). Zhong et al. found that in the psoriasis mouse model, pathogenic NO is produced by NOS2 within local macrophages, and IL-1α is released in the NOS2-dependent manner (Zhong et al., 2018). He et al. also has been reported that psoriasis was characterized by higher levels of expression of innate immunity-related (NOS2/inducible NOS and IL-17C) products (FDR <0.05) (He et al., 2021).

### Vitiligo

Oxidative stress is considered an initiated and mediated factor in the pathogenesis of vitiligo. In vitiligo patients, Li et al. found significant decreases in the levels of antioxidant enzymes (CAT and SOD) and total antioxidant capacity (TAC), while increases in the levels of MDA and 8-OHdG. Moreover, these abnormal indexes about oxidative stress were correlated with activity and disease severity (Li S. et al., 2021). The ROS generator 2,2′-azobis(2-amidinopropane) dihydrochloride (AAPH) treated keratinocyte is a model for studying oxidative stress-mediated skin diseases(Barygina et al., 2019a). Elevated ROS in the skin destroys melanocytes by damaging DNA and related cellular structures, causing the formation of disorders of the melanin. Furthermore, ROS changes the structure of melanocytes and induces many kinds of oxidation products, including oxidated protein products and glycation products (Mitra et al., 2017; Vaccaro et al., 2017). It is believed that the mitochondrial dysfunction caused by oxidative stress is an important cause of melanocyte death. Yi *et al.* showed that inhibition of peroxisome proliferator-activated receptor-gamma coactivator 1-alpha (PGC1-*α*) and enhanced carbonylation in melanocytes of vitiligo could lead to dysregulation of nicotinamide adenine dinucleotide (NAD^+^)-dependent deacetylase Sirtuin 3 (Sirt3, an enzyme involved in suiting mitochondrial dynamics and homeostasis), which led to mitochondrial severe dysfunction and apoptosis of melanocyte (Yi et al., 2019).

Recent studies have shown that RIP1-mediated mitochondrial ROS further triggers the production of necrotic bodies, leading to programmed cell death in melanocytes (Li B. et al., 2021). It suggests that oxidative stress induces various forms of cell death in melanocytes. Moreover, the endoplasmic reticulum stress and the UPR induced by ROS are essential factors causing melanocyte death. The dilation of the endoplasmic reticulum in melanocytes from vitiligo patients has been observed (Boissy et al., 1991). The pathogenesis of endoplasmic reticulum stress in vitiligo remains to be elucidated, but it is speculated that Ca^2+^ interference and translocation of calreticulin (CRT) may be the main pathways leading to melanocyte death. Other studies have shown that the UPR is activated during keratinocyte differentiation (Celli et al., 2011). UPR works in three branches, including PERK, ATF6, and IRE1. The study found that IRE1α/sXBP1 in keratinocytes led to elevated inflammatory factors such as CXCL16, which subsequently mediated CD8^+^ T cell chemotaxis and cytotoxicity. This study also suggests cross-talk between oxidative stress and autoimmune-mediated melanocyte cytotoxicity. Ahn et al. observed that ROS induced ATP release in keratinocyte, and the elevated ATP concentrations produced ROS in melanocytes and activated the ATP/P2X7 receptor-dependent inflammasome. And this stimulated the keratinocyte to produce CXCL9, which recruits CD8^+^ T cells to kill melanocytes (Ahn et al., 2020).

## Gut Microbiome and Inflammatory Skin Diseases

### Gut Microbiome and Gut-Skin Axis Hypothesis

The gut microbiome is the sum of the microbes that contain 3.3 million eukaryotic genes. More than 99% are bacteria. They participate in food digestion and nutrient intake in the human body to maintain human health (Kinross et al., 2011; Dominguez-Bello et al., 2019). Microbiome development and changes are influenced by many factors, including childbirth, diet, drugs, and diseases (Dominianni et al., 2015; Patnode et al., 2019; Shao et al., 2019; Zimmermann et al., 2019). Meanwhile, metabolites such as acetate, propionate, and butyrate, which are fermentation byproducts of the gut microbiota, were essential for intestinal health like providing energy to epithelial cells, enhancing the integrity of the epithelial barrier, carrying out immune regulation, and preventing the invasion of pathogens (Dodd et al., 2017). Growing studies confirm that the gut microbiome plays a crucial role in many human diseases. Moreover, the mechanisms may include intestinal barrier dysfunction leading to bacterial translocation (“Leaky Gut”), gut dysbiosis, related metabolites, induced inflammation, and immune disorders (Fan and Pedersen, 2021).

The concept of the gut-skin axis was first proposed by John H. Stokes and Donald M. Pillsbury in 1930 (John and Donald, 1930). They hypothesized that negative emotional states such as depression and anxiety might alter the gastrointestinal function and normal gut microbiome, leading to increased intestinal permeability and systemic inflammation. Studies have shown that mice fed probiotics significantly improve stress-induced neurogenic skin inflammation compared with untreated mice (Guéniche et al., 2010; Lee J. et al., 2016). Although the mechanism has not been elucidated, the authors explain that the gut-skin axis exists because increased intestinal epithelial permeability will activate T cells destroy the immunosuppressive cytokines and Treg cells, leading to systemic inflammation, which may destroy the skin homeostasis. In addition, studies have shown that gut microbes can use the production of neurotransmitters such as acetylcholine, noradrenaline, and dopamine to communicate with surrounding organs via neuronal pathways (Wang and Kasper, 2014). It has also been suggested that changes in the gut microbiome can lead to increased intestinal permeability, allowing inflammation-related products in the gut to enter the systemic circulation directly (Dodd et al., 2017).

### Gut-Skin Axis in Inflammatory Skin Diseases

Recent studies have shown that the aberrant gut-skin relation can deliver a poor AD progression due to the imbalance of gut microbiota and its signaling versus the skin. (Penders et al., 2006; Lee E. et al., 2016). In AD mice, the related metabolites in the intestinal were significantly decreased, and the levels of IL-25, IL-33, and short-chain fatty acid levels were significantly increased. Meanwhile, intestinal pathogenic colonization bacteria can also cause T cells to transform into Th2-type cells in draining lymph nodes and play an immune function (Nylund et al., 2015; Seite and Bieber, 2015; Schwarz et al., 2017). In a study of a twin cohort, gut dysbiosis was detected in allergic infants and increased *R gnavus* was observed before the onset of allergic manifestations and was associated with respiratory allergies coexistent with atopic eczema (*p* < 0.001) (Chua et al., 2018). Lee et al. also found differences in functional genes related to immune development of AD infants via whole-metagenome analysis (Lee et al., 2018). Recent studies also showed some gut marker microbes in AD patients. A longitudinal study measured the gut microbiome and metabolome functionality of 63 eczema infants between ages 3 weeks and 12 months, and an aberrant developmental trajectory was found in atopic eczema (Ta et al., 2020). Meanwhile, a cross-sectional study among 1,440 children showed that the *α*-diversity of fecal microbiota was associated with a decreased risk of eczema (odds ratio [OR], 0.98; 95% CI, 0.97, 1.00), and *Lachnospiraceae* were associated with decreased risks of eczema (OR range, 0.98: 95% CI, 0.97, 1.00) (Hu et al., 2021). Marrs *et al.* also observed that the SCORing AD index at age 12 months was significantly associated with the abundance of *Clostridium sensu stricto* and *Haemophilus* at enrollment (Marrs et al., 2021). Furthermore, scientists also focus on possible mechanisms that link gut microbes to AD. Nowrouzian *et al.* observed that gut colonization by *S. aureus* strains carrying a certain combination of superantigen and adhesin genes was negatively associated with subsequent development of atopic eczema in a Swedish birth cohort and the FARMFLORA birth cohort (Nowrouzian et al., 2017; Nowrouzian et al., 2019). A study from Gachon University developed a murine model of AD by the repeated epicutaneous exposure of tape-stripped skin to ovalbumin. It revealed that the induction of oral tolerance protects mice from AD-like dermatitis and inhibits the increase in small intestinal eosinophils and dysregulated alterations in the gut microbiome (Um et al., 2021). Combining previous studies of AD mice, it may suggest that gut bacterial strains can provide stimulation and promote maturation of the infant immune system.

At the same time, the “gut-skin axis” may also be closely related to the occurrence and development of psoriasis. In a study involving 54 patients with psoriasis and 27 controls, bacterial DNA was detected in the blood of 16 patients with plaque psoriasis but not in any of the controls, levels of inflammatory markers including IL-1β, IL-6, IL-12, TNF, and IFN-γ were also significantly elevated in all 16 patients (Ramírez-Boscá et al., 2015). According to the researchers, the bacterial DNA may originate from the intestinal cavity, suggesting that the decline of intestinal epithelial integrity is closely related to the pathogenesis of psoriasis. Dellacecca *et al.*(Dellacecca et al., 2020) reported that the vitiligo mouse model administered by oral antibiotics changed the distribution of T cells in the gut and skin and decreased the size of lesions. It strongly suggested that changes in the gut microbiome were associated with vitiligo. Ni et al. identified a significant imbalance in the gut dysbiosis of vitiligo patients by 16 S sequencing (Ni et al., 2020). Bzioueche et al. found that vitiligo patients with skin and gut dysbiosis might be related to mitochondrial damage and autoimmune disorder (Bzioueche et al., 2021). We may have a deeper understanding of the interaction between the gut microbiome and inflammatory skin diseases in the future.

## Mechanistic Insights Into Role of ROS-Stressed Gut Microbiome in Inflammatory Skin Diseases

### Induced Gut Dysbiosis and Oxidative Stress by Diet

Diet is an important determinant of human health. According to an epidemiological survey, 11 million people die from the improper diet every year (GBD, 2017). Early in life, diet (such as human milk oligosaccharides (HMOs)) is involved in the shaping and maturing of the human gut microbiome. Then later in life, the intake of solid food gradually enriches the gut microbiome (Galazzo et al., 2020). Recent high-quality clinical evidence and animal model studies have confirmed the close relationship and interactions between diet and gut microbiome (Asnicar et al., 2021; Yap et al., 2021). It is known that enterotypes (fecal communities cluster) were strongly associated with long-term diets, such as protein and animal fat (*Bacteroides*) and carbohydrates (*Prevotella*) (Wu et al., 2011). Based on emerging evidence, particular gut microbes may predict the response to a particular kind of diet (Kolodziejczyk et al., 2019). Dao *et al.* found that *Akkermansia muciniphila* was considerably associated with a more remarkable improvement in insulin sensitivity and lipid metabolism of obese adults on the calorie-restricted diet (Dao et al., 2016). Moreover, intermittent fasting (voluntarily abstained from drinking and eating for specific periods) also showed positive effects on metabolic diseases (Li et al., 2017) and multiple sclerosis (Cignarella et al., 2018) via gut microbiome.

It has been known that high-fat diets (HFDs) can impair the gut barrier (Bisanz et al., 2019), change gut microbial community structure, and produce related metabolites, which can finally induce cardiovascular disease (Yoo et al., 2021), colon tumor (Yang et al., 2022). Yoo et al. found that a high-fat diet impaired the bioenergetics of mitochondria in the colonic epithelium, enhanced respiration-dependent choline catabolism of *E. coli*, and finally increased levels of circulating trimethylamine N-oxide, which is a potentially harmful metabolite produced by the gut microbiome (Yoo et al., 2021). In return, a randomized human intervention study using a very-low-calorie diet showed that caloric restrict-diet associated with impaired nutrient absorption and enrichment in *Clostridioides difficile* (von Schwartzenberg et al., 2021). Moreover, a healthy Mediterranean-style dietary pattern is associated with a specific gut microbial community, and the protective effect of the Mediterranean diet was significantly associated with decreased abundance of *Prevotella copri* and might improve health (Ghosh et al., 2020; Asnicar et al., 2021). A recent study showed that the ketogenic diet also could alter the human and mouse gut microbiota in a manner distinct from high-fat diets (HFDs) and reduce the levels of intestinal pro-inflammatory Th17 cells (Ang et al., 2020). More on diet, gut microbes, and health may have far-reaching implications.

As the organ in direct contact with the diet, the primary stress in intestinal (mainly intestinal epithelial cells (IECs)) (Awada et al., 2012; Tirosh et al., 2015) is closely related to diet (Wellen and Thompson, 2010; Bourgonje et al., 2020). It has been found that many foods can disturb the redox balance in the gut increase the level of localized/systemic ROS. Eventually, redox balance may initiate redox signal transduction in cells and cause the disease. Rajendran *et al.* found that high doses of iron can lead to oxidative stress in the body (Rajendran et al., 2020). It has been confirmed that a high-fat, high-sugar diet can induce systemic oxidative stress, mainly related to the imbalance of gut microbiome and endoplasmic reticulum stress (Carmody et al., 2015; Wu et al., 2015). A study by Vandemoortele *et al.* has shown that the highly active molecule free malondialdehyde, an *Omega*-3 and *Omega*-6 fatty acid-rich lipid peroxidation biomarker (Ayala et al., 2014), is highly reactive with proteins and DNA and produces a variety of adducts. Meanwhile, it had little effect on its reactivity *in vivo* after ingestion through the digestive tract (Uchida et al., 1997; Vandemoortele et al., 2017). Zhang *et al.* found that the levels of oxidative stress in the blood and carbonyl group increased significantly in the prooxidant diet group (*p* < 0.05) (Zhang et al., 2011). Ge *et al.* also found that mice fed with hyperoxic pork showed increased serum lipopolysaccharide (LPS) levels, down-regulation of tight junction-related genes in the mucosa, and disturbance of cecal microbiota, indicating that particular diet increased not only oxidative stress levels in the body but also caused mucosal barrier damage and gut dysbiosis (Ge et al., 2020).

Although there is no evidence that diet plays a vital role in developing and treating skin diseases, some *in vivo* and *in vitro* studies have developed reliable dietary recommendations based on the fundamental mechanisms, such as abnormal activation of autoimmune, oxidative stress, and gut dysbiosis. In vitiligo, for example, a disorder of redox balance (an increase in hydrogen peroxide) enhances the process by which phenolic chemicals compete with tyrosine to produces reactive quinones. The reactive quinone acts as a covalent binding center of incomplete antigens to tyrosinase and produce new antigens. The micromole (non-cytotoxic) *O*-quinone also gains immunogenicity to induce an immune response (Westerhof and d'Ischia, 2007). Compounds containing naturally occurring plant phenols or polyphenols may contribute to the progression of vitiligo through these mechanisms. Therefore, we can speculate that antioxidant foods may have an inhibitory effect on the development of inflammatory skin diseases. Recent research suggests that dietary regulation, which targets gut dysbiosis, may also have the potential to treat inflammatory skin diseases. A study showed that *P. copri* (significantly decreased in AD children) had been attributed mainly to the high consumption of complex carbohydrates. Furthermore, AD children with high sugar content in their diet had a significantly lower *P. copri* (mean ± SD ratio of 1.04 ± 3.32 vs 0.43 ± 0.52; *p* < 0.0001 in children with lower and higher than median sugar intake respectively), suggested the protection role of diet-related gut microbes(Mahdavinia et al., 2019). Flohr et al. showed a 54% lower risk of flexural eczema on skin examination in the breastfeeding intervention compared with the control group (odds ratio [OR], 0.46; 95% CI, 0.25 to 0.86) (Flohr et al., 2018). The role of the gut microbiome in mediating the particular diet on inflammatory skin diseases warrants further investigation.

### Regulation of Gut Microbiome and Systemic Oxidative Stress

Local oxidative stress in the gut can cause damage to the intestinal barrier structure. Mitochondrial DNA damage in intestinal epithelial cells can lead to excessive ROS production, and the increase of 8-OHdG level can lead to mitochondrial dysfunction, and finally aggravate ROS increase and related oxidative damage, the decrease of tight junctions protein (Claudin-1, Ocudin, and ZO-1) expression and the death of intestinal epithelial cells (Hu et al., 2018). As mentioned in the previous section, an important pathogenic role of gut microbes is bacterial translocation due to structural damage of the intestinal barrier. LPS response is a vital marker signal in this progress. Studies have shown that translocation bacteria and their related products can activate abnormal immune signals in lymph nodes or peripheral blood through LPS, binding to the TLR-24 complex and increasing proinflammatory cytokine and ROS/RNS production (Berg and Garlington, 1979; Wiest and Garcia-Tsao, 2005). Increased ROS/RNS also produced DAMPs (damage-related molecular patterns), which increased inflammation in the body (Lucas and Maes, 2013). Moreover, LPS also increased the expression of oxidative stress-related enzymes. For example, LPS can increase the expression of inducible nitric oxide synthase and form RNS (Iovine et al., 2008). LPS also induces NOX activation, excessive ROS production, and activation of downstream NF-κB pathway (Check et al., 2010; Lin et al., 2011). This high level of oxidative stress in the body, in turn, exacerbates the damage to the intestinal barrier, leading to increased levels of inflammation, which eventually leads to the development of disease.

Also, the specific gut microbe can induce ROS production in intestinal epithelial cells. Jones *et al.* have found that intestinal symbiotic bacteria genus *Lactobacillus* can induce intestinal phagocytes to produce ROS, dependent on NADPH oxidase 1(NOX1) (Jones et al., 2013). Lee *et al.* also demonstrated that gut bacteria induce ROS production by their hosts, which leads to gut inflammation. They found that URA^+^ autochthonous bacteria, such as *G. Morbifer* and *L. Brevis*, can activate the PLC β-duoxros pathway by continuously releasing uracil, which leads to the increase of ROS level and the apoptosis of intestinal epithelial cells (Jones et al., 2013). Local ROS in the gut leads to solid antioxidant activity in gut microbes, mediated by metabolites such as SCFA that inhibit peroxisome and activate the Nrf2 pathway. In summary, gut microbes play a critical role in the redox balance of the gut, which is critical to the health of the gut and even the whole body.

### ROS-Stressed Gut Microbiome and Inflammatory Skin Diseases

We defined the gut microbiome as a ROS-stressed gut microbiome, which exists in oxidative stress. As previously described, local excess of ROS in the gut can be caused by diet ingestion or gut-specific bacteria. There are preliminary findings in the microbiome and inflammatory skin diseases. For example, the detection of intestinal bacterial DNA in the blood of patients with psoriasis may indicate the presence of intestinal barrier dysfunction and bacterial translocation. Further study requires whether patients with other inflammatory skin diseases have similar intestinal characteristics.

As we mentioned above, diet is associated with inflammatory skin diseases. A specific diet can lead to localized oxidative stress in the gut, which disrupts gut barrier function. It can also cause bacterial translocation in the ROS-stressed gut microbiome or induce further ROS production in intestinal epithelial cells, intestinal injury, disturbance of redox balance, and inflammation activation. It is well known that many inflammatory skin diseases are often associated with inflammatory bowel disease. For example, some intestinal diseases such as vitiligo, inflammatory bowel disease (IBD), and celiac disease (CD) are complications (Shahmoradi et al., 2013; Hadi et al., 2020). A 10-years retrospective study of the American population showed that IBD incidence in vitiligo patients was 2.13 times higher than normal IBD (*p* = 0.002) (Hadi et al., 2020). CD is an autoimmune disease that affects the small intestine and is characterized by an autoimmune response to gluten intolerance. The ingestion of gluten can lead to localized inflammation of the small intestine and the progressive development of chronic intestinal malabsorption (Green and Cellier, 2007). In a study of 64 vitiligo patients and 64 controls (Shahmoradi et al., 2013), a case-control study immunoglobulin an (IG) anti endodermal antibody and IgA anti glutaminase antibody, a diagnostic marker for CD, were tested, and two women with vitiligo were found to be seropositive. Some reports also suggest psoriasis (Cohen et al., 2009) atopic dermatitis (Soh et al., 2021) in patients with a higher risk of intestinal disease. In summary, the ROS-stressed gut microbiome may hold the key to pathogenesis in inflammatory skin diseases.

## Novel Therapeutic Approaches for Inflammatory Skin Diseases

### Gut Microbiome-Targeted Therapies

The most important driving force in the study of microbial-disease interactions is clinical translation. Current strategies for regulating gut microbes include dietary interventions, prebiotic, bacteriophages, small molecules, drugs, fecal bacteria transplantation (FMT), and live biological agents (LBPs), et al. (Mahdavinia et al., 2019; Sorbara and Pamer, 2022). Many microbiome-based clinical trials are underway, such as the phase three clinical trial of fecal microbiota transplantation of *Clostridioides difficile* infection (Langdon et al., 2021).

Studies have shown that improving the status of the gut microbiome is beneficial for the treatment of skin diseases. In gut inflammation-related mouse model studies, the skin thickened, hair follicle formation improved, and sebaceous cell production increased in mice through feeding with *Bifidobacterium longum HK003*(Lam et al., 2022). In clinical studies, a lower incidence of skin lesions was also observed in IBD patients receiving probiotic supplementation (Satta et al., 2019). The risk of atopic dermatitis is much lower in children who received probiotics during the neonatal period, and infants who received antibiotics during pregnancy are more likely to develop atopic dermatitis and eczema (Kalliomäki et al., 2001). Manzhalii *et al.* in a clinical study of patients with acne, papules, pustular rosacea, and seborrheic dermatitis treated with NISSLE therapy (*E. coli*). 89% of the patients in the treatment group showed significant improvement or complete recovery of their skin lesions (Manzhalii et al., 2016). Dellacecca *et al.* reported the results of the study of the vitiligo mouse model microbiome, in which oral antibiotics were given to vitiligo mice, and changes in the leukoplakia area and distribution of T cells in the intestinal tract and skin were monitored; the results showed that the leukoplakia area of vitiligo mice decreased significantly after oral administration of antibiotics (Dellacecca et al., 2020).

Evidence of targeted gut dysbiosis treatments for AD is emerging. A meta-analysis of synbiotics for prevention and treatment in atopic dermatitis showed that synbiotics were beneficial for treating AD, particularly synbiotics with hybrid strains of bacteria and for children aged 1 year or older (Chang et al., 2016). Probiotics supplementation is an effective method for treating pathological intestinal microbe colonization, which can significantly improve the intestinal barrier, regulate the immune system’s anti-inflammatory response, and promote the synthesis of anti-inflammatory metabolites. However, another meta-analysis found no evidence suggesting that probiotics make a difference in QoL for patients with eczema (six studies; 552 participants; the standardized mean difference (SMD) 0.03, 95% CI -0.36 to 0.42; low-quality evidence), and probiotics slightly reduced investigator-rated eczema severity scores (24 trials; 1,596 participants) (Makrgeorgou et al., 2018). Boutin *et al.* suggested that supplementation with an LBP, which comprises multiple bacterial genera, might inverse allergic disease manifestations (Boutin et al., 2020). Kwon *et al.* observed that oral administration of *L. sakei* WIKIM30 ameliorated lesion of AD mice and increased the relative abundance of intestinal bacteria through modulation of Th2 response (Kwon et al., 2018). *Lactobacillus paracasei* KBL382 isolated from the feces of healthy Koreans also showed the therapeutic potential for AD (Kim et al., 2020). In sum, it is believed that there will be more and more evidence of gut microbiome-targeted therapies for inflammatory skin diseases in the future.

### Redox-Targeted Therapies

With the development of oxidative stress-related research in inflammatory skin diseases, antioxidant therapy is widely studied. Traditional antioxidant therapy studies include oral or topical treatment of plant/animal biologically active components (polyphenol compounds (Moayyedi et al., 2015; Sangaraju et al., 2021), flavonoid (Ding et al., 2021), *et al.*), macromolecular organic compounds (simvastatin, aspirin (Chen et al., 2021), vitamins (A, D, E), *et al.*(Ponce et al., 2012), and inorganic compounds (molecular hydrogen, palladium, and platinum, et al.). More related antioxidants are still being studied. For example, it has recently been found that Haplopine, a biologically active component of plants, increases the expression of SOD, CAT, HO-1 in a concentration-dependent manner *in vitro* and has excellent therapeutic potential in AD mouse models (Kim et al., 2021). Many inorganic substances have strong antioxidant properties, such as the previously mentioned H_2_S (one of the RSS). However, the treatment of some inorganic compounds needs specific drug delivery conditions to ensure the stability of drugs and the therapeutic effect.

NAHS and GYY4137, two common H_2_S donors, can increase the expression of induced nitric oxide synthase and NO secretion by Akt activation, thus inhibiting ERK activation and reducing vascular endothelial-derived growth factor (VEGF) production (Qabazard et al., 2020; Xu et al., 2021). Yang *et al.* designed a new controlled H_2_S releasing molecule based on the solid anti-oxidation property of H_2_S and used the controlled release of H_2_S for anti-oxidation treatment (Yang et al., 2014). Lin et al. designed microparticles (NaHS@MPs) and *in situ* comprising phase-change material that could sustainably release a gasotransmitter H_2_S for therapeutic effects (Lin et al., 2017). Scientists have also successfully designed some drugs for the stable release of H_2_S, including H_2_S-NSAIDs, Acetyl decylasadisulfide (ADA), organic isothiocyanates, et al. (De Cicco et al., 2016; De Cicco et al., 2017; Martelli et al., 2020; Glanville et al., 2021). It indicates that H_2_S, as a new therapeutic method for inflammatory skin diseases, can further application (Xu et al., 2021). Recent studies have shown that topical hypochlorous acid (HOCl) also can treat inflammatory skin diseases. Jandova et al. found that short-term local HOCl exposure can block UVB-induced skin redox-related gene expression, and the specific mechanism of redox regulation remains to be further studied, may involve genes such as TXNRD2, GSS, SOD3, PRDX5, NQO1, GPX2, HMOX1, and SOD3 (Jandova et al., 2021).

Some endogenous hormones can also be antioxidants to treat diseases, notably melatonin. Melatonin, an endogenous hormone, stimulates necessary antioxidant enzymes such as superoxide dismutase, glutathione peroxidase, and glutathione reductase, protects cell membranes from lipid peroxidation and neutralizes toxic free radicals. There have been many positive discoveries about the antioxidant effects of melatonin (Mauriz et al., 2013; Xia et al., 2020). Furthermore, Verena *et al.* found *in vitro* that oxytocin (OXT), a neuropeptide, reduced the proliferation of dermal fibroblasts and keratinocytes in a dose-dependent manner. Moreover, the OXTR knock induced ROS levels and decreases in glutathione (Deing et al., 2013).

The innovative delivery strategy of antioxidant drugs is a novel area. Local treatment of skin is critical in clinic. Targeting local oxidative stress of skin is a challenge for scientists to construct precise blocking of related signal transmission. As mentioned above, scientists have developed various new methods for stably releasing small-molecule compounds for antioxidant therapy. Nano-drug delivery system is also one of the most effective methods for therapy (Quispe et al., 2021). Some studies have shown that the drug-carrying liposomes, for example, the liposome astaxanthin treatment of AD, are better than free astaxanthin (Lee et al., 2020). Guo et al. reported a constructed curcumin (a kind of flavonoid)-loaded GA-TPGS-modified multifunctional compounds (Cur@GA-TPGS-ES), percutaneous administration to treat psoriasis, solved the problem of low transdermal permeability of curcumin alone (Guo et al., 2021).

Emerging antioxidant therapies include stem cell therapies, biological agents, and polymeric materials. Sah et al. used superoxide dismutase 3 (SOD3) transduced mesenchymal stem cells (MSCs) to treat AD mice. Moreover, they found this approach effectively inhibits the inflammatory response subcutaneously (Sah et al., 2018). Phosphodiesterase-4(PDE4) inhibitors are currently approved for the treatment of psoriasis. Brittany *et al.* have found that PDE4 inhibitors can reduce oxidative stress through the inactivation of the NADPH oxidase (Woodby et al., 2020). Recently, Liang *et al.* also found that PDE4 inhibitors can improve the redox imbalance by reducing ROS and MDA production (Liang et al., 2021). Polymer materials are also a new research direction of antioxidant therapy. Recent work by Zhai et al. (Zhai et al., 2021)showed that the Cold Atmospheric Plasma Activated Hydrogel was able to increase the expression of Nrf2 and decrease the activity of Nitric oxide synthase in a mouse model of vitiligo to increase the resistance of the cell to oxidative stress and immune overreaction. The method has been shown to work well in randomized controlled trials.

## Conclusion

**FIGURE 1:**
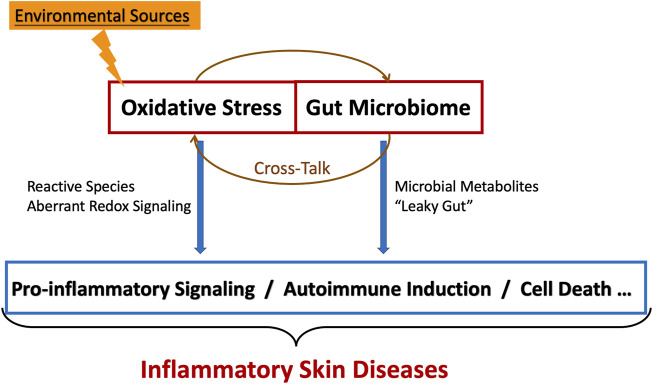
The cross-talk between oxidative stress and gut microbiome in inflammatory skin diseases.

In inflammatory skin diseases, various redox-related signaling pathways mediated by reactive species are involved in cells inducing inflammation and cell death. Research of gut microbiome and skin diseases is still at an early exploration stage. Meanwhile, there is no conclusive evidence for the specific mechanisms between oxidative stress and gut microbiome in inflammatory skin diseases. Our review suggests that the complex microbial-host cross-talk, which occurs through the gut-skin axis, may affect the local/systemic redox status through reactive species, activating local/systemic inflammation, eventually leading to inflammatory skin diseases (Figure 1). The gut-skin axis mediated by the ROS-stressed gut microbiome also provides novel therapies for inflammatory skin diseases.
